# Commentary: Considerations in the Measurement of Glass Transition Temperatures of Pharmaceutical Amorphous Solids

**DOI:** 10.1208/s12249-019-1562-1

**Published:** 2019-12-17

**Authors:** Ann Newman, George Zografi

**Affiliations:** 1 Seventh Street Development Group, PO Box 251, Kure Beach, North Carolina USA; 2 University of Wisconsin-Madison, Madison, Wisconsin USA

**Keywords:** glass transition temperature, wet *T*_g_, dry *T*_g_, glass, differential scanning calorimetry

## Abstract

An increased interest in using amorphous solid forms in pharmaceutical applications to increase solubility, dissolution, and bioavailability has generated a need for better characterization of key properties, such as the glass transition (*T*_g_) temperature. Although many laboratories measure and report this value, the details around these measurements are often vague or misunderstood. In this article, we attempt to highlight and compare various aspects of the two most common methods used to measure pharmaceutical *T*_g_ values, conventional and modulated differential scanning calorimetry (DSC). Issues that directly impact the *T*_g_, such as instrumental parameters, sample preparation methods, data analysis, and “wet” *vs.* “dry” measurements, are discussed.

## INTRODUCTION

### The Amorphous State and Glass Transition Temperature

In recent years, there has been an increased interest in utilizing the amorphous solid form of active pharmaceutical ingredients (API) to overcome the poor aqueous solubility, dissolution, and bioavailability of many corresponding crystalline forms being developed as solid oral dosage forms. Such amorphous API often are combined with amorphous small molecule coformers (1) or polymers (2) to form “miscible” mixtures, which can inhibit solid-state and solution-mediated crystallization during handling, storage, and administration, thus providing greater supersaturation in aqueous solution relative to its crystalline form. As such, it is extremely important to understand the principles underlying the structural, thermodynamic, and kinetic properties of individual API and excipient molecules, and their mixtures, in the amorphous state, and to provide physical measurements that can characterize a particular amorphous system (3).

**Fig. 1:**
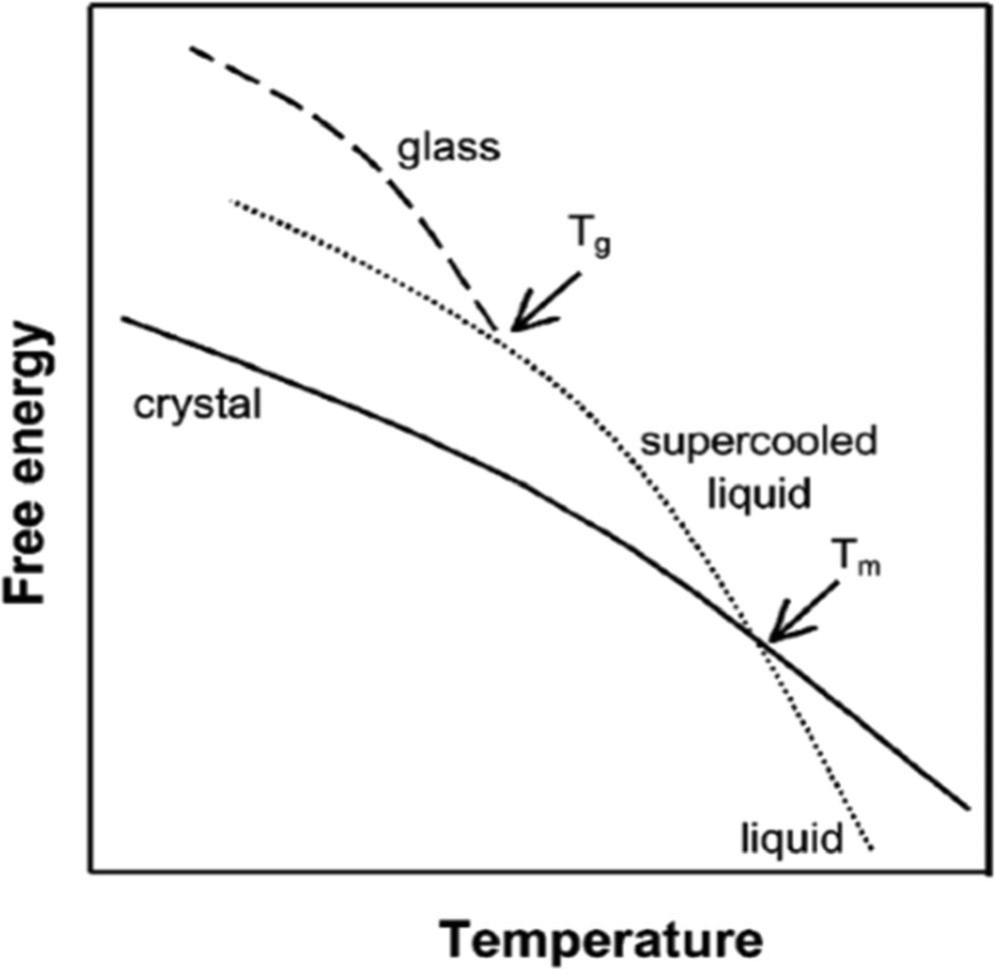
Free energy *vs*. temperature of a molecule reflecting different equilibrium and non-equilibrium states (4)

From a thermodynamic perspective, we can begin by examining in Fig. 1 a schematic representation of the free energy *vs.* temperature profile associated with a typical organic crystal and its corresponding liquid (4). Here, we observe that the free energy of the crystal decreases as the temperature increases until it reaches the melting temperature (*T*_m_). At this point, the free energy of the liquid and crystal are equal and the phase transition from crystal to liquid takes place until only the liquid exists above *T*_m_. If we now slowly decrease the temperature of the liquid phase, under conditions that allow time for crystal nucleation and growth to occur, we see that the crystalline state should return at and below *T*_m_. However, if we decrease temperature below *T*_m_, at cooling rates that do not allow time for crystal nucleation, the free energy of the liquid will increase without the discontinuity ordinarily reflective of some type of phase change. Thus, the super-cooled liquid formed retains the equilibrium properties of the liquid below *T*_m_. However, as also depicted in Fig. 1, the “super-cooled” material maintains the equilibrium properties of the liquid as temperature decreases only until an abrupt discontinuity occurs at a temperature defined as the “glass transition temperature” (*T*_g_). Thus, below this temperature the system is in a thermodynamically unstable “glassy” state, with greater free energy relative to both the super-cooled liquid and crystal. This increased free energy leads to not only greater apparent solubility (supersaturation) than expected for the crystal but also to an increased thermodynamic potential for crystallization and loss of this solubility advantage. It is also thermodynamically likely that molecules in the glassy state under appropriate conditions can lose free energy and approach the super-cooled equilibrium state without crystallizing. This is the basis for the well-described tendency of glasses to undergo “physical aging” below *T*_g_ (5). Thus, it appears important to know whether the operating temperature for various pharmaceutical processes is above or below *T*_g_, *i.e.*, whether one is dealing with the super-cooled equilibrium liquid or the glassy state.

To further understand the underlying molecular basis for the “glass transition,” it should be recognized that while decreasing temperature increases the free energy of the system, the structural viscosity (*η*) of the system, reflective of translational and rotational diffusivity, increases significantly between *T*_m_ and *T*_g_, *i.e.*, from about 10^−2^ Pa at *T*_m_ to 10^12^ Pa at *T*_g_ (6). In addition, studies of the molecular diffusivity of these systems above *T*_g_, have revealed a temperature, *T*_x_, the “crossover temperature” at which the viscosity of the super-cooled liquid begins to change from about 10^2^ Pa at *T*_x_ to 10^12^ Pa at *T*_g_ (6). It is at this temperature, and below, that significant changes in the structure of the super-cooled liquid occur because of the initiation of changes in the arrangements and interactions of molecules, which produce a highly viscous “pre-glassy” state. Once at *T*_g_, the equilibrium super-cooled liquid state cannot be maintained as cooling occurs and viscosity increases, and the thermodynamically unstable glassy state is formed. Thus, the glass transition temperature is not a thermodynamic parameter, but rather, it is a reflection of the diffusive dynamics associated with the changing viscosity as temperature decreases, caused by significant structural changes**.** Therefore, it is not surprising that small differences in values of *T*_g_ determined experimentally at different cooling rates, or by different methods, are often obtained. For example, as a rough “rule of thumb,” a one-order change in the cooling rate produces a 3–5 K change in *T*_g_ (7). In addition to rapid cooling of liquid, very similar amorphous forms, but often with slightly different *T*_g_ values, can be obtained by rapid cooling of molecules in the vapor state, rapid precipitation from solution, mechanical milling of a crystalline sample, and dehydration of some crystal hydrates (8). Consequently, when reporting a value for *T*_g_, it is important to report the exact conditions under which the amorphous solid was formed and the conditions by which *T*_g_ is measured. One additional important reason for knowing the *T*_g_ of any amorphous solid is that it provides a basis for *a priori* estimation of the temperatures at which various important physical events occur. For example, it has been shown that spontaneous crystallization and physical aging, can be inhibited for a period of years *i.e.*, ~ 10^8^ s (900 days) by reducing the temperature to about 50°C below *T*_g_ (9). In addition, it has been established that for most amorphous organic materials and polymers, *T*_x_ is ~ 1.2 *T*_g_ (6), and for many organic molecules, *T*_m_ is ~ 1.5 *T*_g_ (10). Since, it is at this temperature that there is a major change in the heat capacity and thermal expansion coefficient of the system, thermal analytical techniques, such as differential scanning calorimetry (DSC) and dynamic mechanical analysis (DMA), are useful tools for determining *T*_g_ experimentally (3).

### Glass Transition Temperature of Binary Amorphous Mixtures

As mentioned above, amorphous APIs are often mixed with amorphous excipients as part of pharnmaceutical dosage forms. If the two components are completely immiscible and, therefore, phase separated, we would expect the mixture to exhibit two *T*_g_ values each equal to the individual components (11,12). In many cases, small molecule or polymeric excipients can be processed to produce a “miscible” mixture, very much as what one might expect from two miscible liquids of relatively similar chemical structure. Here, we generally observe a single *T*_g_ value dependent on the API-excipient composition and intermediate to the two individual values (11). There are situations where in immiscible systems each of the components have *T*_g_ values which are too close to be detected separately, *i.e.*, within about 10°C, or because the domain size of the separated phases are less than 30–100 nm and not detected by thermal techniques such as DSC (11). In such cases, evidence for the presence of such phase separated domains of individual components has been obtained using X-ray powder diffraction (XRPD) (11) and solid-state nuclear magnetic resonance (SSNMR) spectroscopy (13).

In general, it is known that solutions of two liquids will form if the free energy of mixing is negative. Since the entropy of such mixing is generally positive, leading to a negative free energy, the enthalpy of mixing, arising from intermolecular interactions, will determine whether the mixture is phase separated, or is thermodynamically close to ideal (a regular solution), or non-ideal. Larger enthalpies of mixing, either positive or negative, generally lead to significant non-idealities, and in terms of amorphous solids, we would expect the value of *T*_g_ for a mixture relative to the individual *T*_g_ values, would reflect such thermodynamic differences. For example, it has been been shown that the *T*_gmix_ of an ideal solution will be directly related to the individual *T*_g_ values (*T*_g1_, *T*_g2_) weighted by the weight fraction (*w*) of each component (*w*_1_, *w*_2_) as shown in Eq. (1), the Gordon-Taylor equation (14). Here, temperature must be expressed in Kelvin units.

1 $$ {\mathrm{T}}_{\mathsf{gmix}}=\left\{{\mathrm{w}}_1{\mathrm{T}}_{\mathrm{g}1}+{\mathrm{w}}_2{\mathrm{T}}_{\mathrm{g}2}\right\}/\left\{{\mathrm{w}}_1+\mathrm{K}{\mathrm{w}}_2\right\} $$

where, the constant *K* is,

2 $$ \mathrm{K}=\left\{{\uprho}_1{\mathrm{T}}_{\mathsf{g}1}\right\}/\left\{{\uprho}_2{\mathrm{T}}_{\mathsf{g}2}\right\} $$

Equation (1) has also been derived by Couchman and Karacz (15) on a thermodynamic basis, where,

3 $$ \mathrm{K}=\left(\Delta {\mathrm{C}}_{\mathsf{p}2}/\Delta {\mathrm{C}}_{\mathsf{p}\mathsf{1}}\right) $$

and, ∆*C*_p_ is the heat capacity change for each component at its *T*_g_.

In some cases as with a variety of polymer blends, the densities of the individual compents are close to being equal, so that Eq. (1) can now be written as the Fox equation (16),

4 $$ \left\{1/{\mathrm{T}}_{\mathsf{g}\mathsf{mix}}\right\}=\left\{\left({\mathrm{w}}_1/{\mathrm{T}}_{\mathsf{g}1}\right)+\left({\mathrm{w}}_2/{\mathrm{T}}_{\mathsf{g}2}\right)\right\} $$

This equation is useful as an approximate “back-of-the-envelope” prediction of *T*_gmix_ only knowing the weight fraction and *T*_g_ of each component.

**Fig. 2:**
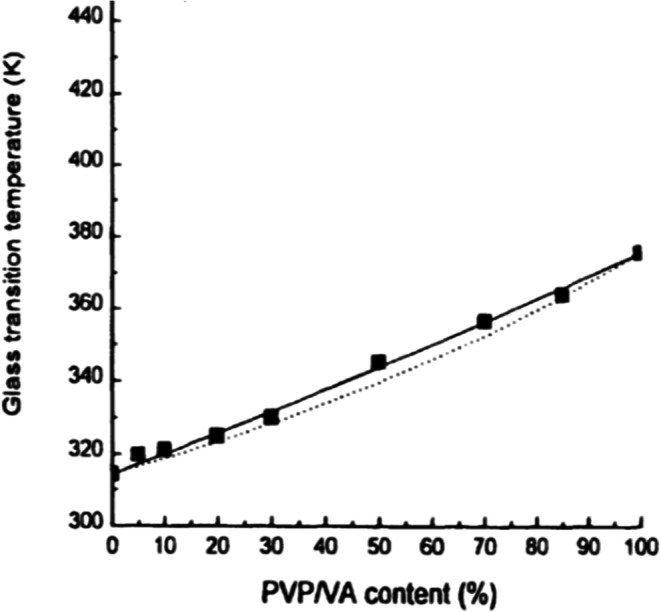
Glass transition temperatures of miscible indomethacin- PVP/VA samples (17):solid line from the Gordon-Taylor equation (14), dotted line from Couchman-Karasz equation (15)

**Fig. 3:**
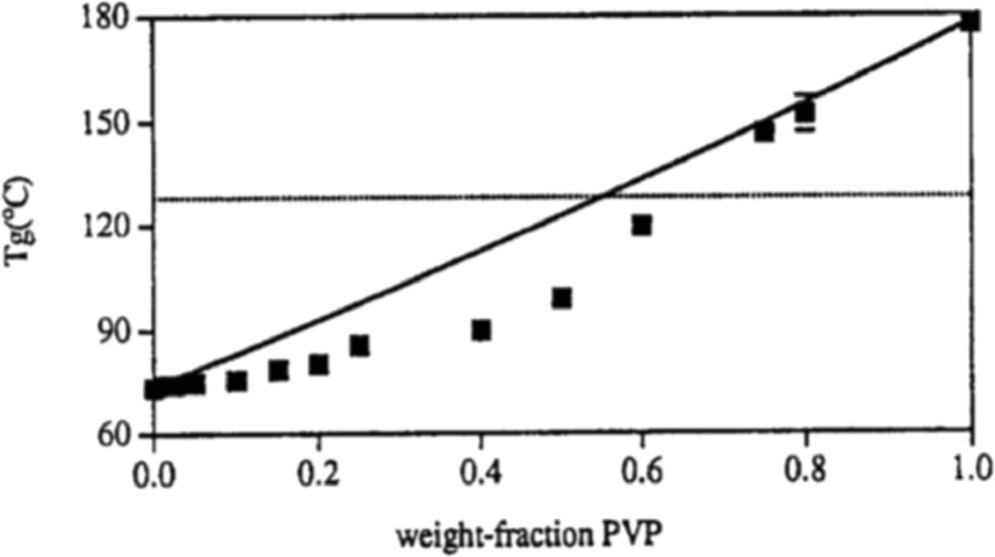
Glass transition temperature of sucrose-PVP amorphous solid dispersions (18); solid line expected from the Gordon-Taylor equation (14)

**Fig. 4:**
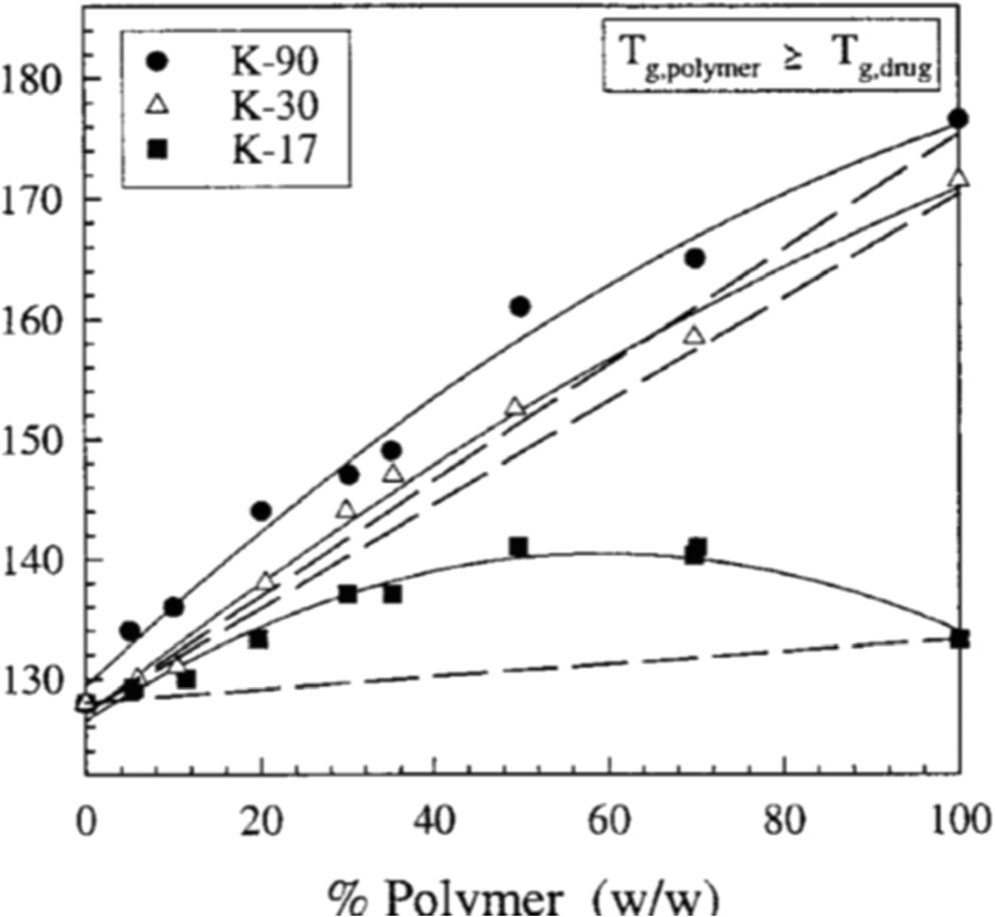
Glass transition temperature of amorphous solid dispersions of MK-0591 with different molecular weight grades of poly(vinylpyrrolidone) (19); dashed lines represent ideal mixing determined by the Gordon-Taylor equation (14)

Generally speaking, if the chemical structures of the individual components are reasonably close, we would expect close to ideal conditions and experimental agreement with the application of Eqs. (1) and (2), as for example, that observed in Fig. 2, for mixtures of indomethacin and PVP/VA (polyvinyl (pyrrolidone)/vinyl acetate, copovidone) (Fig. 2) (17). In those cases where intermolecular interaction between the individual components themselves is greater than that between the two different molecules, we can expect a significant deviation from ideality and experimental values of *T*_gmix_ that are less than predicted by Eq. (1). This type of behavior, illustrated in Fig. 3, has been noted with very polar molecules, such as sucrose, when mixed with PVP, because of the very strong hydrogen bonds that exist between the sugar molecules (18). On the other hand, as shown in Fig. 4, when intermolecular interactions between the components are quite strong relative to bonding of the components with themselves, experimental values of *T*_gmix_ will be greater than predicted. This, indeed, has been reported for amorphous mixtures of an organic API salt and various grades of PVP (19).

### Effcts of Absorbed Water on the Glass Transition Temperature

**Fig. 5:**
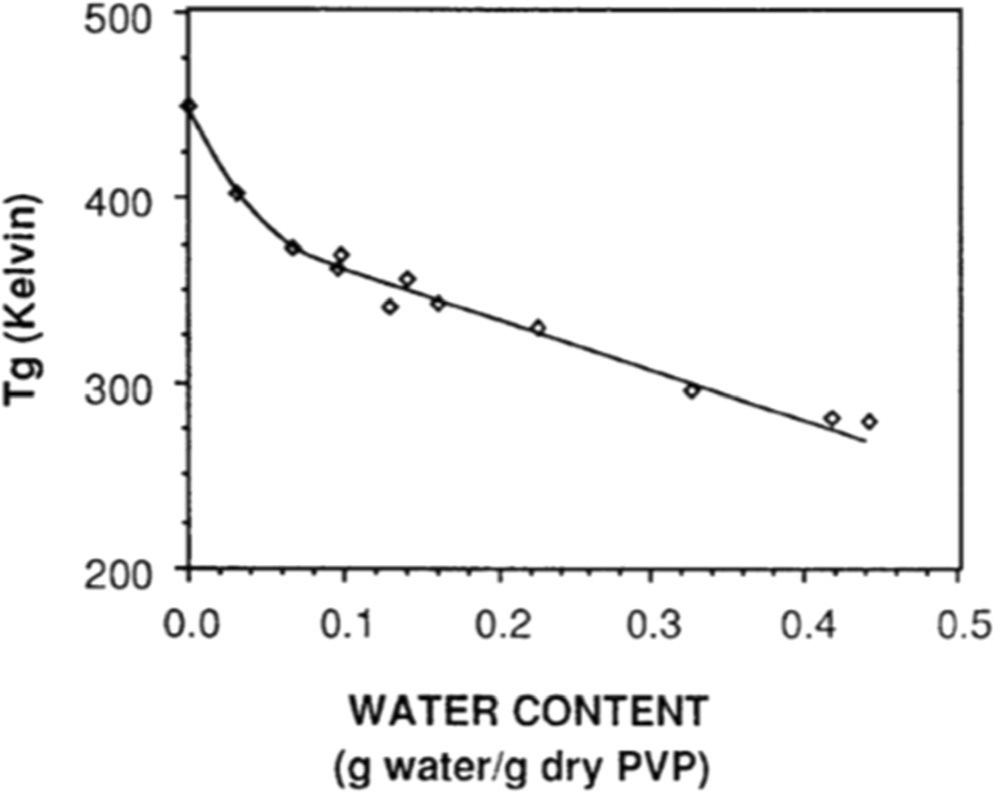
Glass transition temperature of poly(vinylpyrrolidone) as a function of absorbed water content (22)

**Fig. 6:**
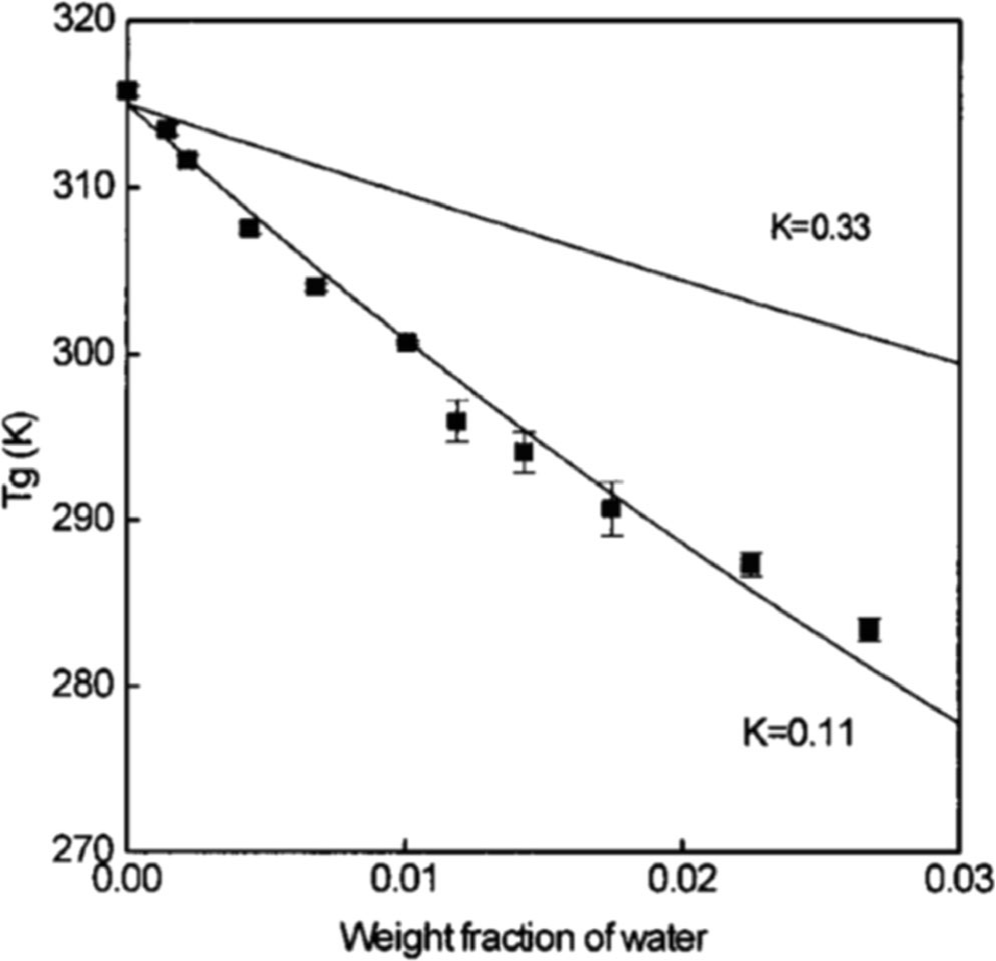
Glass transition temperature of amorphous indomethacin as a function of water content (23); *K* = 0.33 represents ideal mixing from the Gordon-Taylor equation (14), *K* = 0.11 represents non-ideal empirical fit to the Gordon-Taylor equation using experimental data

It is well established that amorphous solid forms of molecules containing hydrogen bond donors and acceptors can absorb significant amounts of water from the vapor state into the bulk phase as a function of relative humidity and temperature (20,21). As such, water molecules essentially dissolve within the glass or super-cooled liquid to form a “solution,” where water molecules break intermolecular hydrogen bonds between the molecules in the solid and form hydrogen bonds with these molecules. Based on the discussion above, it is not surprising that such absorption of water and the formation of a miscible solution within the amorphous form should influence the structure and thermodynamics of the system so as to produce a distinct change in such properties as structural viscosity, free volume, and the glass transition temperature. As observed from the parameters in Eqs. (1) and (4), the very low *T*_g_ of water, 136 K, should greatly reduce the “dry” *T*_g_ of an amorphous solid. Indeed, as shown in Fig. 5, for a PVP-water system, there is a significant reduction in *T*_gmix_ with increasing water content (22). Consider, also, as an example given in Fig. 6, the *T*_gmix_ of an indomethacin-water miscible mixture, produced by exposing amorphous indomethacin to various relative humidities (RH) at 30°C (23). Note that as little as 0.01, weight fraction of water reduces the dry *T*_g_ from 315 to about 296 K, below room temperature. In Fig. 6, we include a plot of data predicted for an ideal mixture by the application of Eqs. (1) and (2) and data obtained experimentally at various water contents. With each plot, we give the *K* constant calculated for ideal mixing from Eqs. (1) and (2), and the *K* constant obtained by allowing it to be an arbitrary fitting constant consistent with the experimental data. Clearly, the absorption of water into indomethacin has a much greater “plasticizing effect, *i.e.*, lower *T*_gmix_, than would be predicted for an ideal mixture. Indeed, this suggests that clustering of water molecules occurs within the relatively nonpolar indomethacin matrix, resulting in a large increase in free volume and much more efficient plasticization than might be expected (23).

Similarly, one would expect water absorbed into a miscible amorphous mixture of API and coformer to reduce the *T*_gmix_ of the binary system, in a manner described above. In such cases therefore, we would expect the absorbed water to produce an increase in molecular mobilty sufficient to bring about physical change unless the operating temperature is significantly less than the resulting *T*_gmix_, *e.g.*, about 50°C below *T*_gmix_. Depending on the strength of the interaction between API and coformer in relation to *T*_gmix_ and the extent of increased molecular mobility caused by absorbed water molecules, a number of scenarios are possible, including: separation into drug-rich and polymer-rich amorphous phases followed by crystallization of drug, or crystallization of drug directly from the miscible dispersion (24,25).

Thus, it is apparent that in studying the properties of amorphous solids it is very important to know exactly what the relationship is between water content and *T*_g_, and how this might change various physical properties. Such understanding will be enhanced if, as a routine, accurate *T*_g_ values are obtained and an appropriate *T*_g_ *vs.* water content profile is experimentally determined.

## *T*_g_ MEASUREMENTS FOR AMORPHOUS SOLIDS

### Techniques Available for Measuring *T*_g_

One of the most common techniques for measuring *T*_g_ is DSC. A modification of this method is modulated DSC (mDSC), which can be more sensitive than conventional DSC. Several other analytical methods have been used to investigate *T*_g_ values, and some of these are summarized in Table I. Minimally, these techniques are not as common as DSC but are becoming more integrated into pharmaceutical laboratories. These methods can be used as confirmation of the value obtained by DSC or as a secondary method if there are issues that make the DSC method difficult or impractical, such as thermal degradation or a lack of sensitivity.

**Table I:** Analytical Methods to Measure Glass Transition Temperatures

Technique	Reference
Scanning force microscopy (SFM)	(26)
Atomic force microscopy (AFM)	(27)
Dynamic mechanical thermal analysis (DMA)	(28–30)
Thermally stimulated current spectroscopy (TSC)	(28)
Dilatometry	(28)
Water diffusion	(30)
Density	(30)
Relative humidity (RH)	(31)
Inverse gas chromatography (IGC)	(32)

In one comparative study, four methods (DSC, DMA, TSC, and dilatometry) were used to measure the *T*_g_ of chitosan (28). The sample preparation and hygroscopicity of the material made analysis difficult, and *T*_g_ values ranging from 150 to 203°C had been reported in the literature. In this controlled study, the four techniques resulted in *T*_g_ values ranging from 140 to 150°C, showing good overall agreement for this compound. For some applications, a range of 10°C may be acceptable, but other applications may need more precise *T*_g_ values, such as determining physical stability conditions and handling conditions when values of *T*_g_ − 50 are close to ambient conditions.

This paper will concentrate on issues to be considered when measuring *T*_g_ values of pharmaceutical samples using conventional and modulated DSC. A number of instrumental and analysis parameters need to be considered when measuring and reporting a *T*_g_ value and these are discussed in more detail in the following sections*.* Before discussing amorphous systems containing water, we will examine the general process of determining *T*_g_ by thermal analysis.

### Experimental *T*_g_ Values Obtained with DSC

**Fig. 7:**
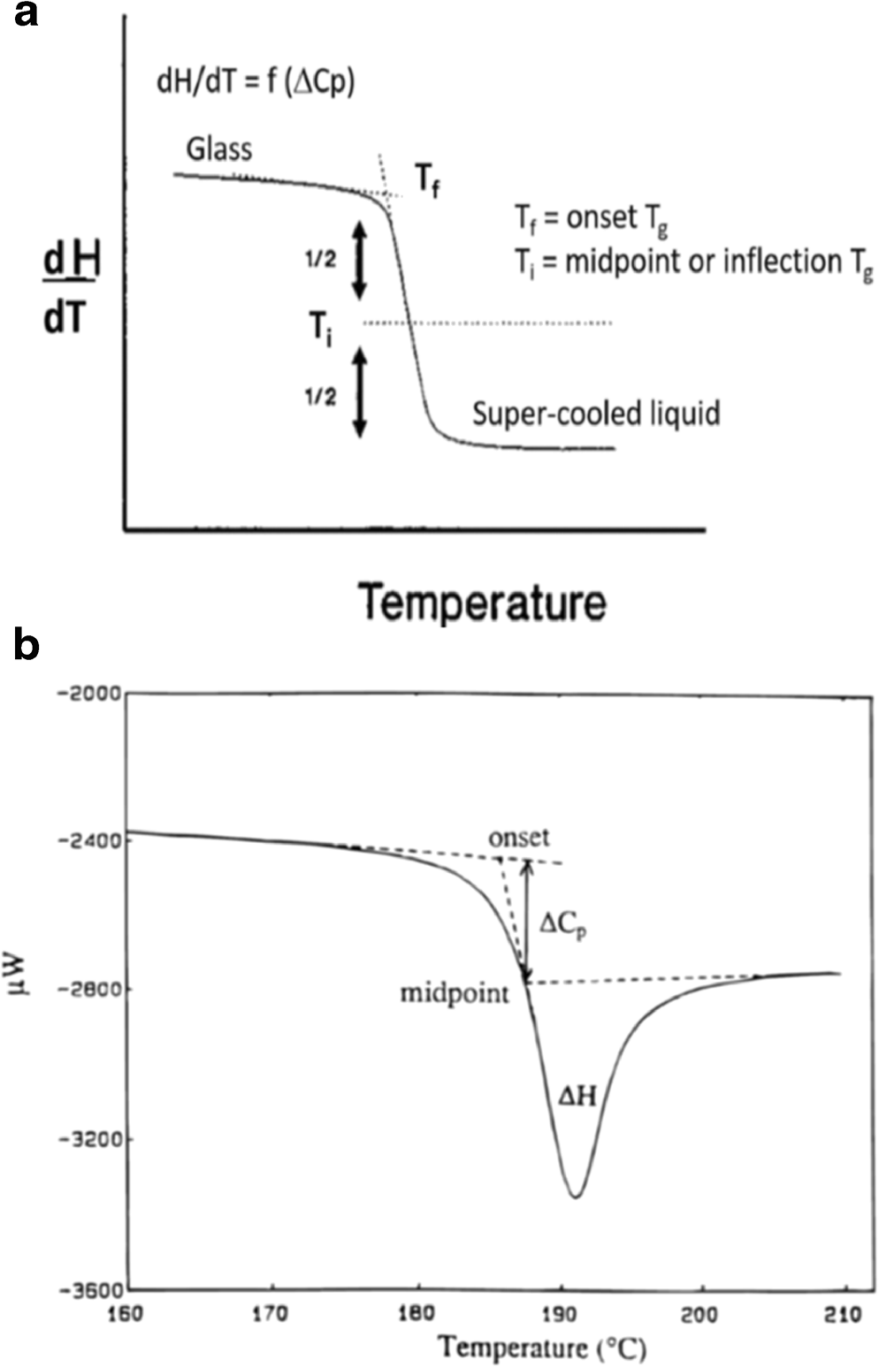
**a** Glass transition in DSC showing onset temperature (*T*_f_) and midpoint (inflection) temperature (*T*_i_). **b** Glass transition temperature showing enthalpy relaxation endotherm (∆*H*) (3)

The glass transition temperature is considered the temperature where an amorphous solid undergoes an apparent second-order transition defined as a step change in the heat capacity (∆*C*_p_) as a function of temperature and observed as a baseline shift (Fig. 7a). This shift represents the change from a glass to a supercooled liquid upon heating (as represented in Fig. 1). There are numerous ways to report the *T*_g_ with the most common being the onset and inflection temperature (Fig. 7a). It is important to understand which temperature is being reported, since they can vary by several degrees, with significant impact on use and storage conditions.

While a classic *T*_g_ signal is represented in Fig. 7a, a small endotherm may also be observed for a glass transition, as shown in Fig. 7b. The small endotherm represents enthalpic relaxation (∆*H*) due to aging or relaxation of the amorphous sample. The enthalpic relaxation endothermic transition will increase as the sample continues to age/relax over time (33). It is possible to minimize/eliminate this endotherm by removing the thermal history of the sample. This involves heating the sample past the *T*_g_, cooling the sample, and reheating the sample past the *T*_g_ (34).

Glass transition temperatures can be used to evaluate the miscibility of amorphous mixtures, such amorphous solid dispersions (ASDs) or polymer blends. When two amorphous materials are miscible, one *T*_g_ will usually be observed in the DSC curve, whereas when the samples are immiscible, two *T*_g_ values will usually be observed (11). The width of the *T*_g_ region can also provide information about the extent of miscibility, *i.e.* broad *T*_g_ transitions indicate tendencies towards immiscibility. An example with miscible polymer blends is given in Table II; as the amount of poly (methylmethacrylate) (PMMA) increases, the *T*_g_ width increases from 26 to 100°C, indicating less miscibility in samples with more PMMA (35). The width was measured by taking the derivative of the *T*_g_ and measuring the onset and final temperature of the derivative peak. Again, it is important to know if the onset or inflection point is being reported when the *T*_g_ signals are very wide. In the case of the 30/70 PECH poly (epichlorohydrin)/PMMA sample, for example, a difference of up to 50°C would be reported for the *T*_g_ onset or inflection temperature.

**Table II:** *T*_g_ Width for PECH/PMMA Polymer Blends (35)

PECH/PMMA^a^	*T*_g_ width (°C)
100/0	20
85/15	26
70/30	65
50/50	80
30/70	100
0/100	40

*PECH*, poly(epichlorohydrin); *PMMA*, poly (methylmethacrylate)

*T*_g_ measurements and relaxation enthalpy can be used to study more complex properties of amorphous materials, such as molecular mobility (4,9,36) and fragility (37). Collecting *T*_g_ values at different heating rates was also used to calculate the “true” *T*_g_ of corn starch containing different amounts of water; this value was the extrapolated *T*_g_ obtained upon regression and represents *T*_g_ when the heating rate is slow and approaching 0°C/min (38).

### Conventional DSC

Conventional DSC instruments measure the heat flow of a sample compared with a reference when both samples are heated with the same controlled temperature program (39,40). Instruments are calibrated with compounds having accurately known melting points and heats of fusion. A common standard used for pharmaceutical applications is indium (melting point 156.6°C, enthalpy of fusion 3.25 kJ/mol). It should be noted that instruments need to be calibrated whenever a different scan rate is employed to ensure that accurate values for temperature and heats of reaction are obtained.

**Fig. 8:**
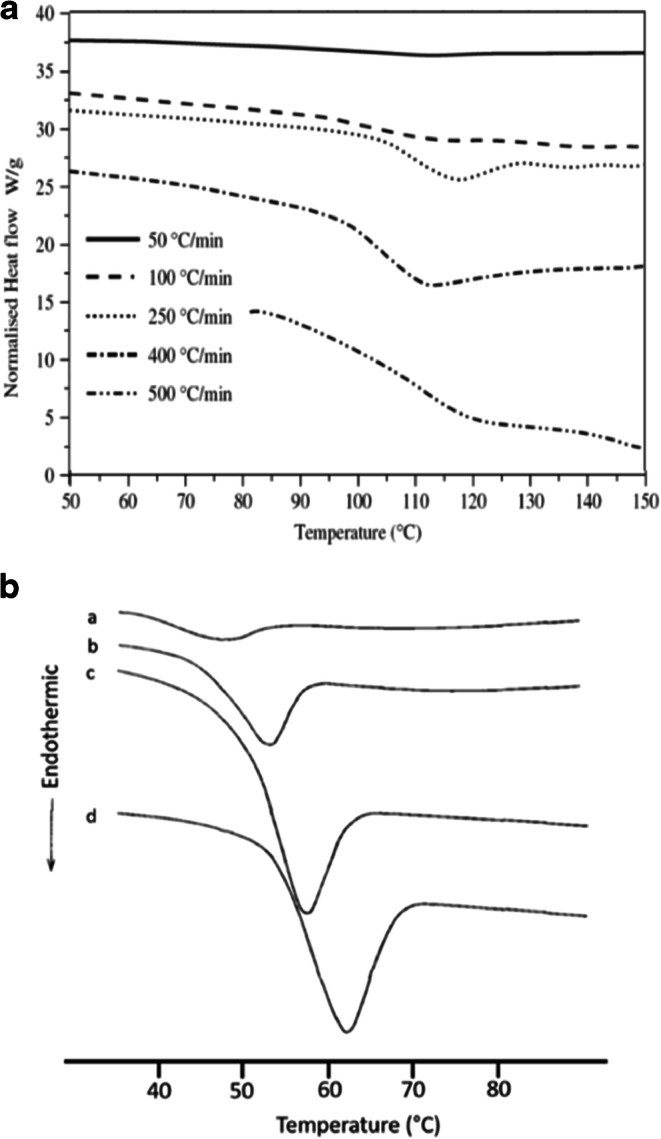
Variation of *T*_g_ with heating rate for **a** amorphous lactose exhibiting *T*_g_ (adapted from (43)) and **b** glassy felodipine with enthalpic relaxation; heating rate: (a) 10.0; (b) 20.0; (c) 40.0; and (d) 80.0 K/min (adapted from (41))

Glass transitions are generally low in energy and can be difficult to see under routine conditions in a conventional DSC. Increasing the scan (heating) rate will typically improve sensitivity and enhance the appearance of *T*_g_ because the flow of energy increases over a shorter period when using a faster scan rate (41–43). This is demonstrated in Fig. 8 for amorphous lactose (43) and glassy felodipine (41) where faster scan rates result in larger signals. Increasing the scan rate will also significantly increase the value of the *T*_g_ temperature, as illustrated with felodipine, which shows a difference of up to 12°C when the heating rate is increased. This example demonstrates the importance of knowing the heating rate when comparing *T*_g_ values since significant differences can be observed.

Thermal events detected by conventional DSC can result because of overlapping transitions, such as *T*_g_ overlapping with enthalpic relaxation, desolvation, or crystallization transitions. Generally, it can be difficult or impossible to separate or eliminate the overlap using conventional DSC, such as preheating the sample to remove solvent or thermal history, without changing the sample that is being analyzed.

### Modulated DSC

**Fig. 9:**
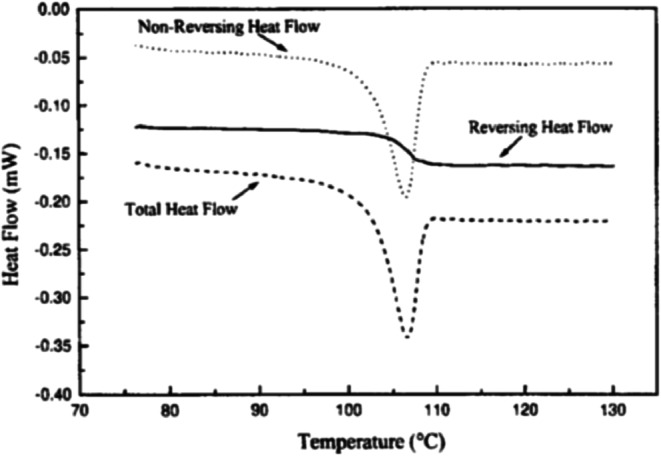
Modulated DSC curves for amorphous saquinavir, showing the reversing, non-reversing, and total heat flow. Note the *T*_g_ in the reversing heat flow curve is separated from the enthalpic relaxation evident in the non-reversing curve (46)

The second technique commonly used for *T*_g_ measurements is mDSC (44,45). It is a technique that uses a combination of a conventional DSC linear heating with a temperature modulation superimposed over the heating rate. The result of this temperature modulation is that the heating rate is no longer constant, but will change in a modulated manner. This allows multiple heating rates to be measured simultaneously, which, in turn, increases resolution and sensitivity, as well as providing a direct measurement of heat capacity (45). It also allows deconvolution of the data into reversing and non-reversing components of thermal events. Reversing transitions include *T*_g_, heat capacity, and melting, while non-reversing events include enthalpic relaxation, desolvation, crystallization, and decomposition. The mDSC plot obtained will contain three curves, including both the reversing and non-reversing curve, as well as a conventional curve, which is similar to that obtained with conventional DSC conditions (Fig. 9).

Most of the mDSC calibration procedures (baseline correction, temperature scale, and sensitivity) are the same as those for conventional DSC using a standard such as indium. An additional calibration for heat capacity is required for mDSC, and sapphire is commonly used for this step. This calibration should be performed using the same modulation program used for analysis (42). For sensitive measurements, it is also important to have sample and reference pans that are the same initial weight to minimize background heat capacities (47), and additional weight corrections may be needed for hermetically sealed pans (48).

**Fig. 10:**
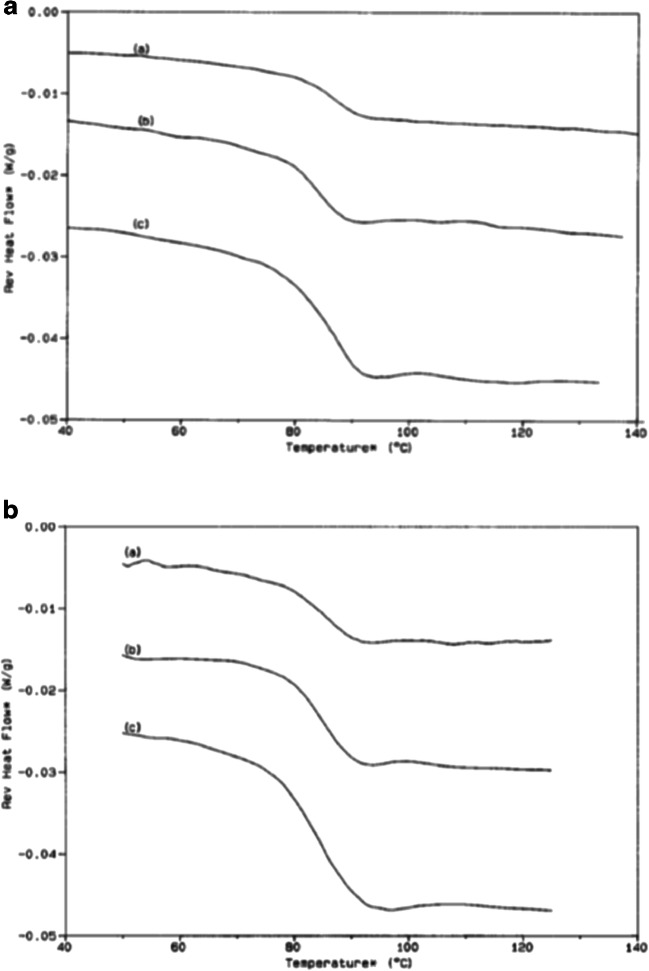
**a** Effect of scan rate on the reversible heat flow of polystyrene; amplitude 1°C over a period of 60 s with a scan rate of (a) 2°C/min, (b) 3.5°C/min, and (c) 5°C/min. **b** Effect of modulation period on the reversible heat flow of a polystyrene sample; scan rate of 5°C/min, amplitude 2°C, and a period of (a) 45 s, (b) 60 s, and (c) 100 s (50)

Several parameters need to be optimized for the mDSC run, including heating rate, modulation amplitude, modulation period, and sample size (45,49). Slow heating rates, ranging from 0.1 to 5°C, are commonly used for mDSC, resulting in longer run times compared with conventional DSC. As observed with conventional DSC, a faster scan rate results in more sensitivity (Fig. 10a). It is suggested that there should be at least five modulation cycles during the transition, therefore the width of the *T*_g_ will impact the modulation amplitude chosen. Modulation periods of 20–80 s are acceptable for many samples, and, as shown in Fig. 10b, the modulation period will influence the sensitivity of the measurement. Sample sizes of 2–20 mg are commonly tested to find the optimal weight for the analysis (45). It should be noted that all parameters need to be optimized for the sample being analyzed (50). A standard set of conditions will not work for all materials, and artifacts due to improper deconvolution can emerge in the reversing and non-reversing curves when the wrong parameters are used. These artifacts could lead to misinterpretation of the data, and, ultimately, affect the use and stability of the amorphous sample during development. Additional information on setting instrumental parameters will be specific to the mDSC equipment being used and can be obtained from the manufacturer.

It has been suggested that “mDSC should be viewed as a complimentary approach to conventional DSC, rather than a replacement. Rather, the best approach for characterizing new materials is to start with conventional DSC and then switch to mDSC if its advantages are required” (45). Collecting the conventional DSC curve under appropriate conditions for *T*_g_ values can also provide useful information when determining mDSC parameters, such as approximate temperature range and transition width, while the mDSC can provide more sensitivity and additional data (such as the heat capacity) if needed for the system being studied.

### Wet *vs.* Dry *T*_g_ Measurements

In this section, some of the unique issues that arise when attempting to measure the *T*_g_ values of an amorphous system when it contains a specific amount of absorbed water will be examined. It is important to understand the type of data required when designing the thermal experiments, and, to assure that the water content in the sample is maintained during the measurement. As summarized in the “INTRODUCTION,” amorphous materials can contain water that may significantly decrease the *T*_g_ of the sample. Collecting data with water contained in the sample is described as a “wet” *T*_g_. In some cases, a *T*_g_ without the water is needed, and this is commonly called a “dry” *T*_g_. It is important to understand the type of data needed in order to pick the appropriate sample preparation and instrumental conditions.

Wet *T*_g_ measurements can be collected using conventional or modulated DSC instruments. There are numerous examples in the literature demonstrating the effect of water on the wet *T*_g_ of amorphous samples, excipients, and dispersions (22,38,51–54). For wet *T*_g_ measurements, the sample pan is hermetically sealed to retain the water in the sample. Crimped, pinhole, and open pans should not be used to determine wet *T*_g_ values. It is important that a true hermetic seal is obtained, since any break in the seal will lead to water loss and an inaccurate *T*_g_ temperature. If significant water is contained in the sample and a large sample size is used, pressure buildup in the pan may be an issue, leading to deformed pans, sample leakage, and reduced contact with the cell, which can result in poor reproducibility and potential artifacts in the data (43). It has been reported that the integrity of the seal can be an issue above approximately 150°C (55). Smaller sample sizes can help reduce pressure buildup and its issues when using routine pans or commercial high-pressure pans (~ 150 Bar) are available from instrument vendors. An easy test to confirm that the seal is intact is to weigh the sample after the run and confirm that no change in weight has occurred when compared with the initial sample weight (55). Small sample sizes in a hermetically sealed pans can also cause issues. It has been reported, for example, that water loss into the headspace can result in a dehydration endotherm when using hermetically sealed pans with small amounts of hydroxypropylmethylcellulose (HPMC) films. Weighing the pan after the run showed no loss in weight, indicating the water was retained in the pan (56). Note that the dehydration temperature might be slightly higher due to the more confined area in the DSC pan. Increasing the sample size in the pan, using an inverted lid, or using a smaller pan will minimize or prevent this from occurring. Other considerations, such as contact of the sample with the sample pan and baseline corrections, may also be needed for certain compounds (57).

For dry *T*_g_ measurements using a conventional DSC, the sample can be dried before the analysis or during the run. If it is dried before the analysis, it is important to understand how exposure to ambient conditions during DSC sample preparation may change the water content of the sample, and this preparation method is not recommended for hygroscopic samples. A second method to collect dry *T*_g_ values is to use an open pan, collect data at ~ 20°C/min to a temperature above the *T*_g_ (to erase thermal history and remove water or volatiles), quench cool to 50°C below the *T*_g_, hold for about 10 min, and then reheat through the *T*_g_ (34). A third cycle through the *T*_g_ is recommended. If the *T*_g_ is reproducible in the second and third cycles, then the transition is confirmed as a *T*_g_. If different values are obtained in the second and third cycles, then the transition may not be a true *T*_g_ and other characterization of the sample may be needed. The use of a dry purge gas, such as nitrogen, in the DSC instrument should maintain a dry environment throughout the heat/cool process.

**Fig. 11:**
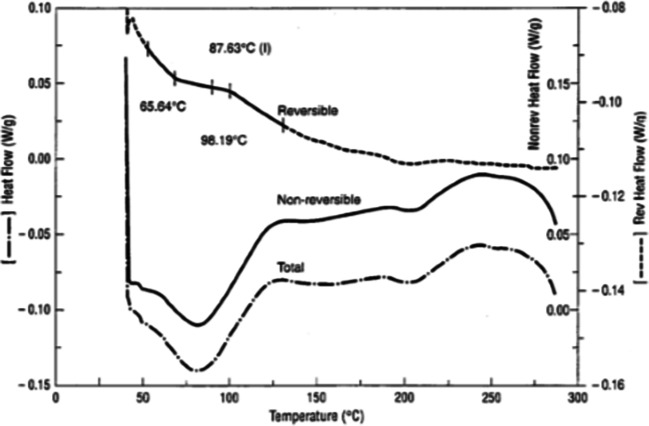
A polymeric drug substance showing the loss of water in the broad non-reversible endotherm and the underlying *T*_g_ in the reversible curve (59)

An open pan can also be used to collect dry *T*_g_ measurements using mDSC. Crimped or pinhole pans will allow egress of water out of the pan, but the rate of dehydration will not always be consistent, due to the variable extent of crimping or size of a manual pinhole in the lid, and this inconsistency could significantly affect the thermal curves observed (49,58). If mDSC parameters are chosen correctly, the *T*_g_ obtained should be separated from the water loss, with the *T*_g_ in the reversing curve and the dehydration in the non-reversing curve, as shown in Fig. 11. Multiple cycles with mDSC have also been reported to remove thermal history (46), and multiple mDSC heating cycles could also be used to confirm dehydration and the dry *T*_g_.

## CASE STUDIES

### Amorphous Citric Acid

A study with citric acid, a common excipient used in lyophilization and oral dosage forms, was performed to investigate the wet and dry *T*_g_ of the amorphous material (60). Conventional DSC data were collected, using scan rates of 10 K/min, and additional studies were performed using scan rates of 5–40 K/min. Amorphous samples were made by heating and quenching the crystalline materials in pinhole (anhydrate) or hermetically sealed (monohydrate) pans and subsequently measuring the *T*_g_. KF values of the crystalline materials resulted in less than 0.05% water in the anhydrate and 8.6% water in the monohydrate.

**Fig. 12:**
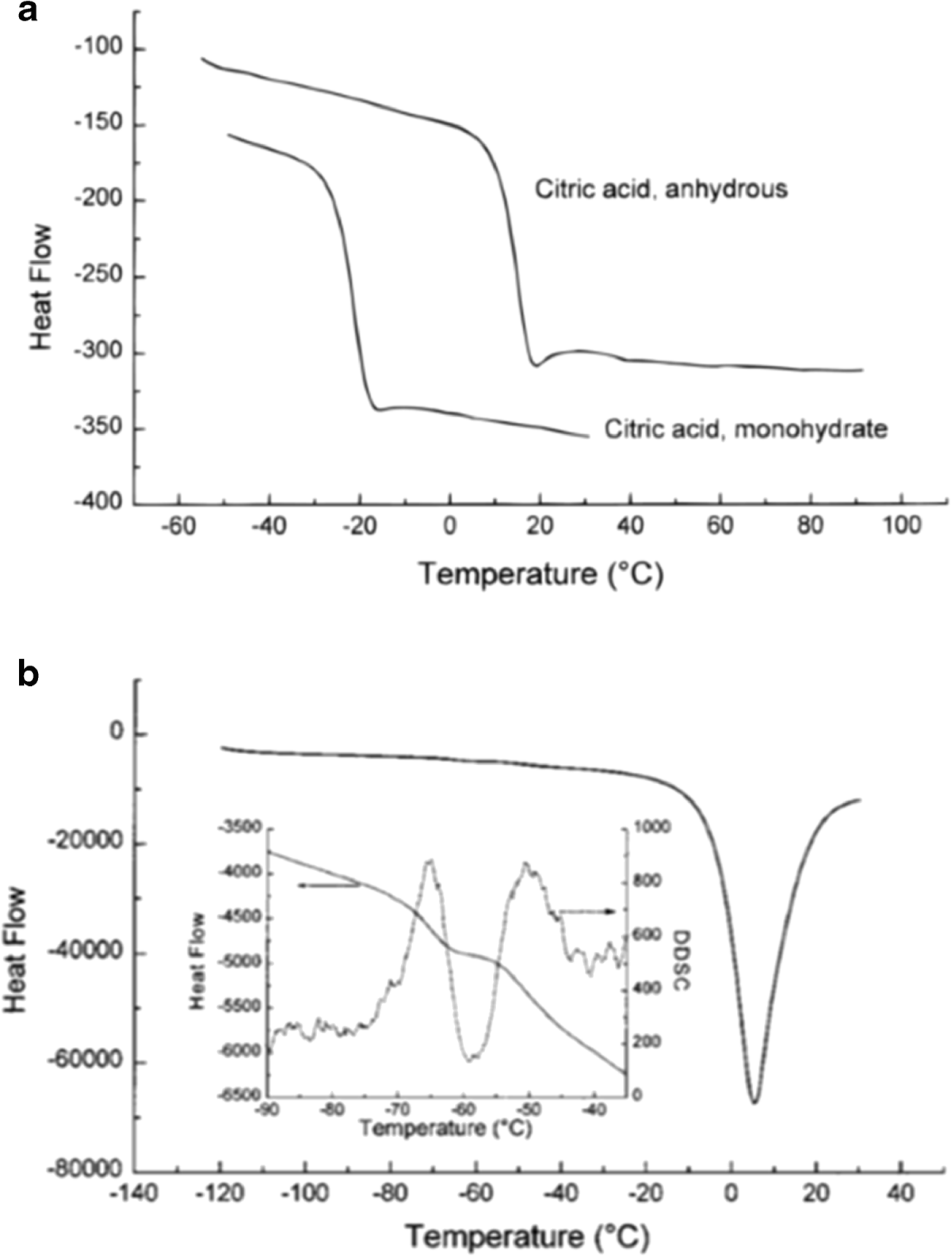
DSC curves for **a** dry (anhydrous) and wet (monohydrate) amorphous citric acid samples showing the *T*_g_ values for each sample; **b** 10% (*w*/*v*) citric acid aqueous frozen solution. Insert shows two thermal events between − 90 and − 40°C. The derivative DSC (DDSC) curve clearly shows two transitions (60)

The *T*_g_ value measured for the dry citric acid was 11°C, while the wet citric acid (containing 8.6% water) was significantly lower at − 25°C (Fig. 12a). An additional experiment was performed with the monohydrate in a pinhole pan, which resulted in *T*_g_ and ∆Cp values similar to the dry amorphous citric acid material, indicating that the pinhole pan resulted in loss of water during the DSC experiment. The low *T*_g_ values explained the difficulties encountered trying to produce and maintain these amorphous materials when made by traditional methods, such as quench melting on a larger scale. The addition of water sorbed by the amorphous material under ambient conditions, thereby lowering the *T*_g_ even further, added to the stability issues. The Gordon-Taylor equation was used to calculate the *T*_g_ for mixtures of citric acid and water. Excellent agreement was found between the calculated and experimental values. This analysis allowed accurate estimations for the *T*_g_ of amorphous citric acid water mixtures for water contents up to 8.6% water.

The DSC curve for a 10% (*w*/*v*) citric acid aqueous frozen solution (Fig. 12b) exhibited two thermal events between − 90 and − 40°C. The lower temperature is attributed to the *T*_g_ of a freeze-concentrated solution with high water content but without ice, and the higher transition was due to a freeze-concentrated solution containing ice and is represented as *T*_g_′. For successful freeze drying, the sublimation of ice should be at or below the *T*_g_′. In this case, the *T*_g_′ of citric acid solutions was substantially lower than − 45°C, the lowest temperature that could be attained with their laboratory equipment, therefore, producing amorphous citric acid by lyophilization without crystallization was not successful.

### Immiscibility in Amorphous Solid Dispersions with Water

The phase behavior of amorphous solid dispersions containing the hydrophilic polymer PVP and various hydrophobic drugs (nifedipine, indomethacin, ketoprofen, droperidol, and pimozide) was investigated after exposure to elevated RH conditions at room temperature (24). Samples were analyzed using DSC and infrared (IR) spectroscopy to determine miscibility. For all initial ASD samples, analysis confirmed complete miscibility by DSC (one *T*_g_) and IR (specific drug-polymer interactions).

**Fig. 13:**
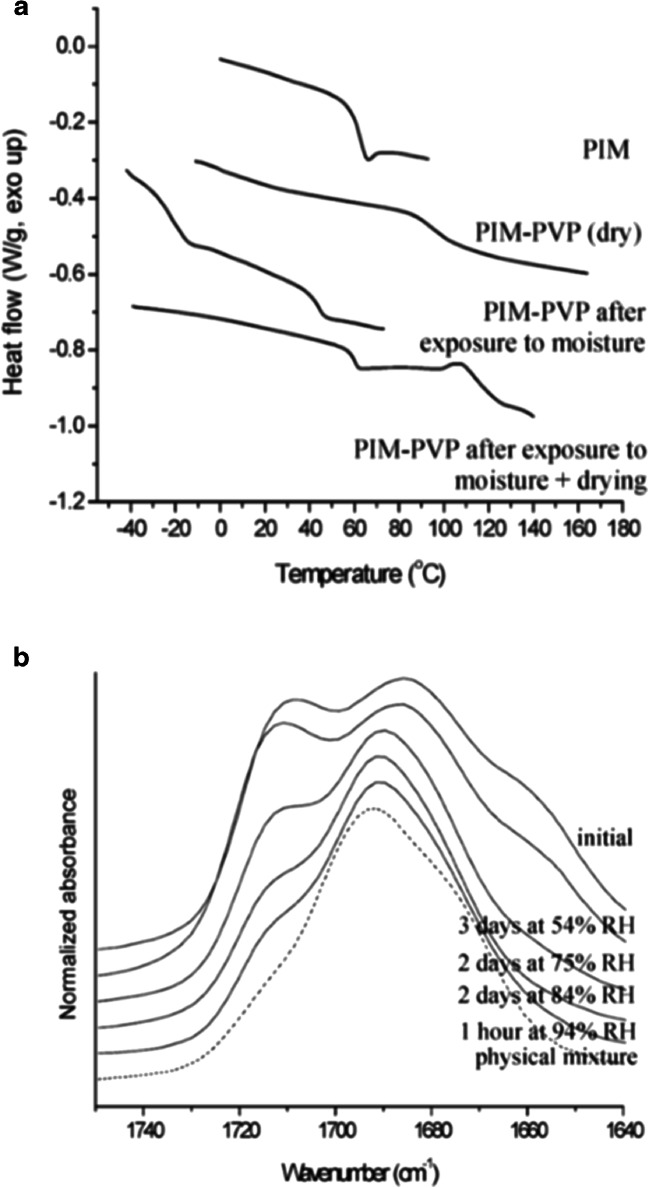
Analysis of pimozide-PVP ASDs before and after RH exposure and subsequent drying **a** *T*_g_ values before and after exposure to 94% RH with and without drying. The *T*_g_ of pure amorphous pimozide (top) is included for comparison. **b** IR spectra of the carbonyl regions for pimozide-PVP ASD. A reduction in the intensity of peaks assigned to the free pimozide carbonyl (2709 cm^−1^) and PVP carbonyl when hydrogen bonded to pimozide (1661 cm^−1^) was observed as the storage RH was increased and the resulting spectra were found to be more similar to the calculated spectrum of the physical mixture composed of amorphous drug and PVP (24)

Storage at elevated RH conditions (75–94% RH) resulted in two different phenomena. Two dispersions (indomethacin-PVP and ketoprofen-PVP) were found to maintain miscibility after water exposure. The other three systems were found to separate into immiscible drug-rich and polymer rich amorphous phases, in a process called amorphous-amorphous phase separation (AAPS). As shown in Fig. 13a, for the pimozide-PVP system, the dry *T*_g_ of the amorphous pimozide was reported as 60°C (dry PVP K12 was reported around 112°C). The DSC curves clearly showed one *T*_g_ initially (94°C) for the ASD, while two *T*_g_ values were evident after exposure at 94% RH for 42 h (− 21 and 44°C). The marked decrease in both *T*_g_ values, compared with the initial *T*_g_ values for the individual components, was due to the water (22.4%) absorbed by the ASD. Upon drying the samples (after RH exposure), two *T*_g_ values are still evident, but are now reported at higher temperatures (61 and 112°C). The sample remains a mixture, but the *T*_g_ values of the individual components increase due to the water lost upon drying.

The IR spectra confirmed the separation of the pimozide-PVP system. After RH exposure the relative intensity of the peak at 1709 cm^−1^ (assigned to free carbonyl moiety of the drug) was found to decrease, which suggested an increase in the drug-drug hydrogen bonding (Fig. 13b). A similar decrease was observed in the intensity of the peak at 1661 cm^−1^, which was assigned to the carbonyl group of PVP when it is hydrogen bonded to drug molecules. These observations were consistent with phase separation of the system into drug- and polymer-rich amorphous regions and confirmed the DSC results.

Physical stability and crystallization were also studied for the various ASDs. Drug crystallization in systems exhibiting AAPS was found to occur earlier (< 6 days at 94% RH) when compared with systems that remain miscible (> 46 days at 94% RH). Evidence of water-induced phase separation was observed after storage at RHs as low as 54% for the pimozide-PVP system. It was evident that the drug crystallization was much faster following the AAPS, since the crystallization inhibitory influence of the polymer would be reduced. These studies highlight one issue with accelerated stability testing at high RH conditions, which may not be representative of physical stability that could be achieved at lower RH or with protective packaging to limit exposure to water.

## CONCLUSIONS

As with any solid-state characterization method, it is important to understand the instrumental parameters and sample preparation methods used to collect *T*_g_ data. The choice of conventional *vs*. modulated DSC instruments will be dependent on the amorphous system being analyzed, and, while mDSC can help improve sensitivity and separate overlapping transitions, it may not be the best choice for all samples. Other parameters, such as sample pan configuration and scan rate, can drastically change the value and should be specified for all reported *T*_g_ values. When collecting data, it is important to understand if the effect of water is required, and parameters should be chosen to optimize the “wet” *T*_g_ value. When reporting “wet” *T*_g_ values, it is important to provide accurate water values. When reporting dry *T*_g_ values, it is imperative to remove all water in the samples to provide an accurate value.

## Abbreviations

AAPS: Amorphous-amorphous phase separation
AFM: Atomic force microscopy
API: Active pharmaceutical ingredient
ASD: Amorphous solid dispersion
*C*_p_: Heat capacity
DSC: Differential scanning calorimetry
DDSC: Derivative differential scanning calorimetry
DMA: Dynamic mechanical analysis
*H*: Enthalpy relaxation
HPMC: Hydroxypropylmethylcellulose
IGC: Inverse gas chromatography
kJ/mol: Kilojoule/mol
mDSC: Modulated differential scanning calorimetry
mg: Miligram
nm: Nanometer
η: Structural viscosity
Pas: Pascal seconds
PECH: Poly (epichlorohydrin)
PMMA: Poly (methylmethacrylate)
PVP: Polyvinyl (pyrrolidone)
PVP/VA: Polyvinyl (pyrrolidone)/vinyl acetate, copovidone
RH: Relative humidity
SFM: Scanning force microscopy
SSNMR: Solid-state nuclear magnetic resonance
*T*_f_: Onset temperature
*T*_g_: Glass transition
*T*_g_′: Glass transition due to freeze-concentrated solution containing ice
*T*_g1_: Glass transition of component 1
*T*_g2_: Glass transition of component 2
*T*_i_: Inflection temperature
*T*_m_: Melting temperature
*T*_gmix_: Glass transition of mixture
TSC: Thermally stimulated current
*T*_x_: Crossover temperature
*w*_1_: Weight fraction of component 1
*w*_2_: Weight fraction of component 2
XRPD: X-ray powder diffraction
